# Change in Prevalence of Hypertension among Korean Children and Adolescents during the Coronavirus Disease 2019 (COVID-19) Outbreak: A Population-Based Study

**DOI:** 10.3390/children10010159

**Published:** 2023-01-14

**Authors:** Kyungchul Song, Se Yong Jung, Juyeon Yang, Hye Sun Lee, Ho-Seong Kim, Hyun Wook Chae

**Affiliations:** 1 Department of Pediatrics, Severance Children’s Hospital, Endocrine Research Institute, Yonsei University College of Medicine, Seoul 03722, Republic of Korea; 2 Division of Pediatric Cardiology, Department of Pediatrics, Yonsei University College of Medicine, Seoul 03722, Republic of Korea; 3 Biostatistics Collaboration Unit, Yonsei University College of Medicine, Seoul 03722, Republic of Korea

**Keywords:** hypertension, COVID-19, child, adolescent

## Abstract

During the coronavirus disease 2019 (COVID-19) outbreak, the prevalence of obesity increased globally; this may be associated with hypertension incidence. However, investigations on the changes in the prevalence of hypertension among children and adolescents are limited. This cross-sectional study investigated the prevalence of hypertension among 1428 youths aged 10–18 years using data from the Korea National Health and Nutrition Examination Survey 2018–2020. We assessed the prevalence of hypertension according to sex, age, body mass index (BMI), and residential district. The prevalence of hypertension increased from 7.1% to 12.5% in all participants. In the sex-specific analysis, the prevalence was found to be increased in boys. In the age-specific analysis, the prevalence was found to be increased in youths aged 13–15 years. In the BMI-specific analysis, an increase in the prevalence was prominent in the normal BMI group. In the residential district-specific analysis, the prevalence of hypertension among youth increased in urban areas. Our results show that the prevalence of hypertension increased among Korean children and adolescents during the COVID-19 outbreak. These findings suggest the importance of close monitoring of hypertension among youth during the COVID-19 pandemic.

## 1. Introduction

Children and adolescents seem to be less affected by the global coronavirus disease-2019 (COVID-19) pandemic than adults, constituting approximately 2% of the affected cases [1] and reporting less severe symptoms [2]. Nevertheless, the COVID-19 pandemic has significantly impacted their lives. Many governments adopted social restriction policies, including lockdowns, social distancing, and school closures, to mitigate the spread of the virus [3], which in turn caused an increase in cardiometabolic risk [4]. In South Korea, the first case of COVID-19 was confirmed on 20 January 2020. Schools were closed from March through May 2020, and a stepwise on-site school reopening began in June 2020 [5]. Social distancing and the shutdown of educational activities have affected children’s lifestyles. Decreased physical activity [6], longer screen-based sedentary time [7], and changes in nutritional habits [8] caused an increase in obesity and metabolic syndrome, even in children [9]. Obesity is a well-known risk factor for hypertension [10], is related to hypertension severity, and is a predictor of morbidity in adulthood [11]. The incidence of obesity was increasing in children and adolescents before COVID-19 but has accelerated during the pandemic for the reasons mentioned [9].

Hypertension is a risk factor for COVID-19 infection and severity [12]. The Health Outcome Predictive Evaluation of COVID-19 Registry reported increased mortality among COVID-19 patients with and without arterial hypertension at 29.6% and 11.3%, respectively [13], with increasingly diverse complications. Furthermore, children with hypertension are vulnerable to obesity and metabolic syndrome due to COVID-19 preventive restriction policies [14]. Additionally, variants in the angiotensin-converting enzyme 2 (*ACE2)* gene found in obese adult patients with hypertension has been proposed as mechanisms for severe COVID-19 infection [15]. This close relationship between obesity, hypertension, and COVID-19 is interesting, especially considering the long-term cardiovascular risk of hypertension in children [16]. Nevertheless, investigations on the changes in the prevalence of hypertension during the COVID-19 pandemic among Korean children and adolescents are limited.

This study aimed to investigate changes in the prevalence of hypertension among Korean children and adolescents according to sex, age, body mass index (BMI), and residential district during the COVID-19 outbreak using data from the National Health and Nutrition Examination Survey (KNHANES).

## 2. Materials and Methods

### 2.1. Study Design and Participants

This study analyzed data acquired from KNHANES, a national surveillance system based on the National Health Promotion Act and executed by the Korea Centers for Disease Control and Prevention since 1988. KNHANES uses a two-stage systematic stratified sampling method for a cross-sectional survey conducted in urban and rural settings and includes 17 administrative districts. Data is collected from health interviews, examinations, and nutritional evaluations of a nationally representative population, with assessments of health-related behaviors, anthropometric measurements, biochemical and clinical profiles, and nutritional evaluations [17]. Among the 23,461 participants included in the KNHANES from 2018 to 2020, 1582 were aged 10–18 years. After excluding participants with diabetes mellitus and/or missing anthropometric and/or blood pressure data, 1428 children and adolescents were included in this study. Participants with diabetes mellitus included those with known diabetes, fasting glucose level ≥ 6.9 mmol/L, and/or hemoglobin A1c level ≥ 6.5% [18].

### 2.2. Study Variables

This study collected data regarding age, sex, anthropometric measurements, and residential district. Height was measured to the nearest 0.1 cm using a portable stadiometer (range, 850–2060 mm; Seriter, Holtain Ltd., Crymych, UK), and weight was measured in the upright position to the nearest 0.1 kg using a calibrated balance beam scale (Giant 150N; HANA, Seoul, Republic of Korea). BMI was calculated as weight (kg) divided by height in meters squared (m^2^). Waist circumference (WC) was measured midway between the costal margin and iliac crest at the end of normal expiration, and the waist-to-height ratio (WHtR) was calculated as WC (cm)/height (cm). Height, weight, and BMI were presented as standard deviation score (SDS) values from the 2017 Korean National Growth Charts [19]. Children were classified as normal (<85th percentile), overweight (85th to <95th percentile), or obese (≥95th percentile) according to their BMI. We assessed the proportion of abdominal obesity using two definitions: (1) WC > 90th percentile using the Korean waist reference [20], and (2) WHtR > 0.5 [21].

Residential districts were classified into rural and urban areas based on the administrative district and population. In Korea, city levels are named Si, Gu, and Gun, and district levels are named Dong, Eup, and Myeon, respectively. Si and Gu are urban areas with large populations and usually consist of Dong, whereas Gun is a rural area with a smaller population and consists of Eup and Myeon. We defined Dong as an urban area and Eup and Myeon as rural areas.

Blood pressure (BP) was measured on the right arm supported at the level of the heart after the participants had been seated for 5 min of rest using a mercury sphygmomanometer (Baumanometer sphygmomanometer W.A. Baum Co Inc., Copiague, NY, USA, and Littmann Stethoscopes; 3M, Maplewood, MN, USA) with an appropriately sized cuff. The appropriate cuff size was defined as an inflatable bladder width that was at least 40% of the arm’s circumference at a point midway between the olecranon and acromion and a length covering 80–100% of the arm’s circumference. Trained healthcare professionals (nurses and technicians) measured the participants’ BP three times, according to a standardized protocol. The first (K1; the first appearance of sound) and fifth (K5, the disappearance of sound) Korotkoff sounds represented the systolic BP (SBP) and diastolic BP (DBP), respectively, and the mean SBP and DBP were calculated as the average of the second and third readings. Hypertension was defined as SBP and/or DBP ≥ 95th percentile according to age- and sex-specific reference standards for Korean children and adolescents [22]. High SBP was defined as an SBP ≥ 95 percentile, and high DBP was defined as a DBP ≥ 95 percentile.

### 2.3. Laboratory Analysis

Blood samples were collected from an antecubital vein following an 8 h fast and were subsequently processed and immediately refrigerated. The serum levels of fasting glucose, total cholesterol, high-density lipoprotein cholesterol (HDL-C), and triglycerides were measured using Labospect 008AS (Hitachi, Tokyo, Japan). Low-density lipoprotein cholesterol (LDL-C) levels were calculated using the Friedewald formula (LDL-C = total cholesterol − [HDL-C + (triglycerides/5)]). This formula was used for serum samples with triglyceride values ≤4.5 mmol/L, whereas LDL-C was set as missing for samples with triglyceride levels >4.5 mmol/L because the formula loses its accuracy when the triglyceride level is above 4.5 mmol/L [23]. Dyslipidemia was defined as one or more abnormal levels of any lipid profile according to the pediatric guideline [24].

### 2.4. Statistical Analysis

All continuous variables were expressed as weighted means with standard errors, whereas categorical variables were expressed as weighted percentages with standard errors. Participants were divided into subgroups according to BMI, age, sex, and residential district. The changes in the proportion of participants with obesity, abdominal obesity, and hypertension between 2018, 2019, and 2020 were analyzed. The differences between groups were tested using analysis of variance for continuous variables and the Rao-Scott Chi-square test for categorical variables. Logistic regression analysis was performed to show the odds ratio of year for hypertension after adjusting for age, sex, BMI, and WC. All statistical analyses were performed using SAS version 9.4 (SAS Institute, Cary, NC, USA) for the complex survey design, with clustering, stratification, and unequal weighting of the KNHANES sample. All *p*-values were calculated using two-tailed *t*-tests, and a *p*-value < 0.05 was considered statistically significant.

## 3. Results

### 3.1. Characteristics of the Participants According to Sex

**Figure 1:**
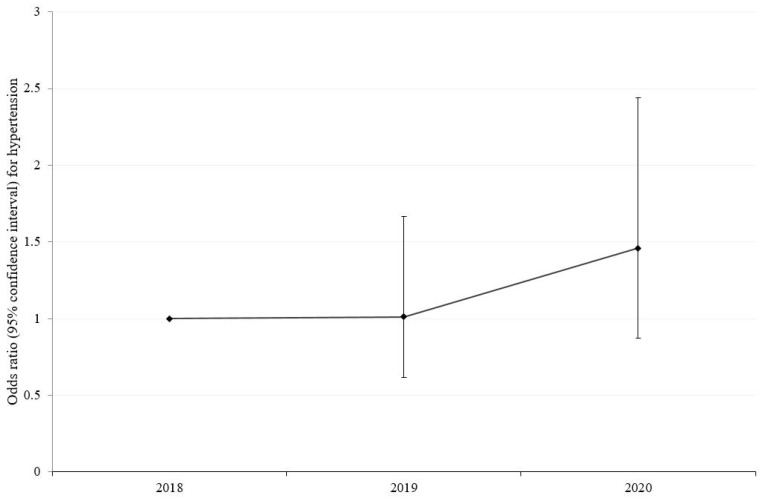
Odds ratio (95% confidence interval) of year for hypertension after adjusting for age, sex, BMI, and WC. BMI, body mass index; WC, waist circumference.

WC (*p* = 0.006), WHtR (*p* = 0.001), and the proportion of participants with WHtR > 0.5 (*p* = 0.036) increased from 2018 to 2020 (Table 1). Additionally, DBP (*p* = 0.018) and the proportion of participants with hypertension (*p* = 0.016) also increased during this period (Table 1). In logistic regression, the odds ratio of hypertension in 2020 was not significantly higher when compared to those in 2019 after adjusting for age, sex, BMI, and WC (Figure 1).

In sex-specific analyses, during 2018–2020, WC (*p* = 0.023) and WHtR (*p* = 0.006) increased among males, as did SBP (*p* = 0.044), whereas DBP was found to have increased among females (*p* = 0.008; Table 2). The proportion of participants with hypertension increased only in males (*p* = 0.036).

### 3.2. Prevalence of Hypertension According to Age

In the age-specific analysis, during 2018–2020, the proportion of overweight and obese participants increased in the age group of 13–15 years (*p* = 0.006) (Table 3). WC (*p* = 0.034), WHtR (*p* = 0.015), and the proportion of participants with WHtR > 0.5 (*p* = 0.025) increased in the age group 13–15 years, while WHtR increased among the participants aged 16–18 years (*p* = 0.026). DBP increased significantly among participants aged 13–15 years (*p* = 0.033). The number of participants with high SBP (*p* = 0.007) or DBP (*p* = 0.009) increased in the age group 13–15 years. The proportion of participants with hypertension increased significantly only among the participants aged 13–15 years (*p* < 0.001).

### 3.3. Prevalence of Hypertension According to BMI

In the BMI-specific analyses, during 2018–2020, the WHtR increased in the normal (*p* = 0.028) and overweight (*p* = 0.008) groups (Table 4). The proportion of participants with a WC > 90th percentile increased in the obesity group (*p* = 0.031), while those with a WHtR > 0.05 increased in the overweight (*p* = 0.047) and obesity (*p* = 0.044) groups. DBP increased significantly in the normal group (*p* = 0.018). The proportion of participants with hypertension increased significantly only in the normal group (*p* = 0.041).

### 3.4. Prevalence of Hypertension According to Residential District

In the residential district-specific analyses, WC and WHtR increased among participants in both urban and rural areas (*p* = 0.034 for WC in urban areas, *p* = 0.009 for WHtR in urban areas, *p* = 0.021 for WC in rural areas, and *p* = 0.028 for WHtR in rural areas) (Table 5). The proportion of participants with a WC > 90 percentile (*p* = 0.013) and those with a WHtR > 0.5 (*p* = 0.003) increased among the participants in rural areas. DBP (*p* = 0.019) and the proportion of participants with hypertension (*p* = 0.031) increased in urban areas.

## 4. Discussion

This study showed an increased prevalence of hypertension and abdominal obesity among Korean children and adolescents during the COVID-19 pandemic. In addition, there was a statistically significant increase in WC and WHtR, although the change in the mean values was small. An increase in the prevalence of hypertension and abdominal obesity was prominent in the year 2020 compared to 2018 and 2019, as COVID-19 had not yet influenced the health status of Koreans. The increase in the prevalence of hypertension and abdominal obesity was more prominent in boys than in girls and in youths aged 13–15 years than in other age groups. In the BMI-specific analysis, an increase in the prevalence of hypertension was prominent in the normal BMI group. Additionally, an increase in the proportion of patients with hypertension was more apparent among youths in urban areas than among those in rural areas.

The present study detected a prevalence of arterial hypertension in Korean adolescents of 7–8% in the years just before the SARS-CoV-2 pandemic. In a previous study among Korean children using KNHANES 2007–2015, the prevalence of hypertension before the COVID-19 era was reported to demonstrate a gradual increase from 6.9% to 9.0% [25], higher than that reported in other studies [26,27]. A recent meta-analysis including 47 articles with 186,630 participants reported a pooled prevalence of 4.0% for pediatric hypertension [27]. This discrepancy could be for the following reasons: (1) Our study included children aged 10–18 years, in whom hypertension is more prevalent than in younger children [27]; (2) a mercury sphygmomanometer was used for BP measurement in the KNHANES, which also showed a higher prevalence of hypertension [27]; (3) BP measurements were not repeated at different visits, whereas the diagnosis of hypertension needs three separate visits [28,29]. Another meta-analysis also revealed that the prevalence of elevated BP decreased at repeated visits (12.1%, 5.6%, and 2.7% during the first, second, and third visits, respectively) [26]. So, the prevalence of hypertension might have been overestimated. However, it remains that, by consistently using the same KNHANES screening measurement, it increased significantly during the COVID-19 outbreak.

The increase in the prevalence of hypertension (up to 12.5%) without a significant increase in the prevalence of obesity in our study might be associated with increases in WC, WHtR, and abdominal obesity and the impact of COVID-19 infection. Pediatric hypertension is associated with not only obesity but also abdominal obesity [30]. Moreover, abdominal obesity was associated with hypertension even after adjusting BMI in a population-based study [31]. In addition to the role of generalized and abdominal obesity in hypertension during this pandemic era, COVID-19 infection is a risk factor for developing hypertension. A prospective multicenter study revealed that COVID-19 was positively associated with high SBP and DBP among children and reported the proportion of stage-1 hypertension as 11% [32]. An altered *ACE2* axis was suggested to be related to the pathogenesis of hypertension in that study. Further studies are required to clarify the relationship between COVID-19 and pediatric hypertension.

Obesity is strongly associated with the prevalence and severity of hypertension in children and adolescents [33]. Furthermore, it is known that approximately one out of three youths have two or more cardiovascular risk factors [30]. Our study demonstrated that hypertension is more prevalent in obese and overweight children and adolescents than in those with normal weight, which was concordant with the results of previous studies. The German-Austrian-Swiss obesity registry demonstrated a significantly higher prevalence of hypertension, triglycerides, and LDL-C among overweight and obese children and adolescents compared to normal-weight participants [10]. The prevalence of hypertension was 6.1%, 22.0%, 29.9%, and 44.5% in the normal-weight, overweight, obese, and extremely obese groups, respectively. A KNHANES-based study showed that the prevalence of hypertension increased from 14.9% in 2007–2009 to 27.7% in 2013–2015 among obese youths and from 6.3% in 2007–2009 to 7.4% in 2013–2015 among non-obese youths [25]. Even before the COVID-19 outbreak, the prevalence of obesity was found to increase over time [34]. However, the prevalence increased dramatically during the COVID-19 pandemic: from 23.4% to 27.4% in the US [35] and from 10.5% to 12.3% in Israel [36]. This increase might be associated with decreased physical activity and increased screen-based sedentary behavior due to social distancing during the COVID-19 pandemic period [7].

In our study, increases in the prevalence of hypertension were more prominent among youths with normal BMI. This might be associated with factors related to hypertension which were not investigated in this study, such as physical activity and sleep pattern. Qiu et al. reported that children’s blood pressure increased independently of BMI during the COVID-19 outbreak, which might be due to environmental factors such as excessive screen time and poor sleep [37]. In addition, a decrease in physical activity due to social distancing might be more prominent in children with normal BMI compared to those with obesity because the baseline physical activity of obese children might have been lower before the COVID-19 outbreak. Further studies considering BMI and environmental factors associated with hypertension are required to clarify this increase in the prevalence of hypertension in children according to BMI during the COVID-19 outbreak.

This study showed that the prevalence of hypertension was higher in boys than in girls, as observed in previous research [25,27]. Additionally, the increase in the prevalence of hypertension, along with increases in WC and WHtR, was more prominent in boys. This might be because the change in physical activity during the COVID-19 outbreak was more pronounced in boys, considering the differences in behavioral characteristics according to sex. A Korean study reported that physical activity was reduced in boys but remained constant in girls during the COVID-19 outbreak [38]. In girls, diastolic BP increased during the COVID-19 outbreak without a change in the prevalence of diastolic hypertension. The long-term outcome of diastolic hypertension in children is not fully understood, but a recent North American study showed that children with isolated diastolic hypertension have distinct clinical features such as leaner body habitus and fewer cardiovascular risks [39]. Further studies are required to clarify this phenomenon.

The prevalence of hypertension increased significantly only in middle school students (aged 13–15 years), which might be related to altered body composition. In this group, the WC, WHtR, and the proportion of WHtR > 0.5, and overweight and obese participants increased. This distinct phenomenon could be explained by the following: (1) School closure was implemented more strictly in middle schools than in high schools in Korea; (2) the COVID-19 vaccine was administered to 18-year-old youths earlier than young children and gradually applied to younger ages; thus, the duration of school closure and home study was longer in middle school students than in high school students; (3) compliance with protective recommendations against COVID-19 such as social distancing was different among various age groups [40]. High school students showed higher physical and social activity than middle school students. Further studies to clarify the effect of the COVID-19 outbreak on the prevalence of hypertension according to age are required, using data from 2021 and 2022, which would minimize the effect of school closure.

In our study, an increase in the prevalence of hypertension was prominent in urban areas, whereas the prevalence of hypertension and obesity was higher in rural areas. Previous studies reported that hypertension was more prevalent in rural areas [41] due to a higher proportion of overweight and obese individuals and a lower resting metabolic rate in both children and adults [41,42]. Additionally, a cross-sectional study reported that living in a rural area is a risk factor for obesity and high BP, which might be related to a carbohydrate-based diet [43]. The effects of COVID-19 on obesity in urban and rural areas in previous reports have been controversial [37,44]. A Chinese study reported that the risk of high BP decreased among children in rural areas, and the prevalence of high BP was lower in rural areas than in urban areas during the COVID-19 outbreak [37]. A cross-sectional study reported that changes in BMI among adults were not associated with the location of residence [44]. The significant increase in the prevalence of hypertension seen only in urban areas might be associated with the following: (1) Urban areas have dense populations [45]. Thus, the impact of COVID-19 on hypertension might be higher in urban areas compared to rural areas; (2) environmental factors, including sleep patterns and screen time, might contribute to this difference [37], and (3) larger sample sizes in urban areas compared to rural areas might have contributed to this statistically significant difference. Further studies investigating the regional differences in cardiometabolic risk associated with COVID-19, including hypertension, are required.

This study has some limitations. First, this was a cross-sectional study of the Korean population alone. Second, white-coat hypertension and secondary hypertension were not excluded because ambulatory blood pressure monitoring and hormone and imaging studies were not included in the KNHANES. As mentioned previously, the blood pressure values were obtained in a single visit, although three measurements were performed, and the dominant arm was not considered in the measurement. Third, environmental factors associated with hypertension, such as physical activity, screen time, sleep patterns, and dietary modification, were not analyzed. Finally, the sample size of rural areas was smaller compared to those of urban areas. Despite these limitations, this study showed an increase in the prevalence of hypertension during the COVID-19 outbreak among children and adolescents, further analyzing the results according to age, sex, BMI, and residential characteristics using nationally representative data.

## 5. Conclusions

This study showed the prevalence of hypertension in children and adolescents during the COVID-19 pandemic by evaluating multiple parameters. The increase was apparent in boys, middle school students, youths with a normal BMI, and those residing in urban areas and might be related to the impact of COVID-19 on hypertension and an increase in central obesity among youth. Further studies investigating changes in the prevalence of hypertension during the COVID-19 outbreak and associated risk factors may be interesting and potentially useful in order to establish preventative strategies for pediatric hypertension.

## Figures and Tables

**Table 1:** Baseline characteristics of participants.

Variable	2018	2019	2020	*p*
Total number, *n*	488	529	411	
Age, y	14.4 (0.1)	14.3 (0.1)	14.2 (0.2)	0.544
Sex (male), %	51.5 (2.5)	52.4 (2.6)	54.4 (2.7)	0.730
Residential district (urban), %	90.8 (2.8)	85.0 (3.6)	90.7 (2.8)	0.318
Height SDS	0.30 (0.06)	0.31 (0.06)	0.32 (0.07)	0.988
Weight SDS	0.17 (0.06)	0.24 (0.08)	0.31 (0.08)	0.391
BMI SDS	0.02 (0.07)	0.10 (0.09)	0.19 (0.08)	0.262
BMI percentile				0.306
Normal, %	80.5 (2.0)	75.8 (2.8)	75.2 (2.4)	
Overweight, %	7.9 (1.3)	8.4 (1.5)	11.0 (1.7)	
Obesity, %	11.7 (1.7)	15.8 (2.3)	13.8 (2.0)	
WC, cm	70.3 (0.5)	71.8 (0.7)	72.9 (0.7)	0.006
WC > 90 percentile, %	10.5 (1.5)	12.80 (1.9)	15.5 (2.0)	0.157
WHtR	0.43 (0.00)	0.44 (0.00)	0.45 (0.00)	0.001
WHtR > 0.5, %	11.7 (1.5)	16.7 (2.4)	18.9 (2.1)	0.036
SBP, mmHg	108 (1)	109 (1)	109 (1)	0.098
DBP, mmHg	67 (1)	68 (1)	69 (1)	0.018
SBP > 95 percentile, %	3.8 (0.92)	3.9 (0.93)	5.5 (1.0)	0.380
DBP > 95 percentile, %	5.1 (0.98)	5.1 (1.2)	8.2 (1.7)	0.146
Hypertension, %	7.1 (1.1)	8.2 (1.3)	12.5 (1.8)	0.016
Glucose, mmol/L	5.1 (0.02)	5.1 (0.02)	5.1 (0.03)	0.354
Hemoglobin A1c, %	5.3 (0.01)	5.4 (0.02)	5.3 (0.02)	0.007
Total cholesterol, mmol/L	4.3 (0.03)	4.3 (0.04)	4.3 (0.04)	0.807
HDL-cholesterol, mmol/L	1.3 (0.01)	1.4 (0.01)	1.3 (0.02)	0.028
LDL-cholesterol, mmol/L	2.5 (0.03)	2.5 (0.04)	2.5 (0.03)	0.442
Triglycerides, mmol/L	1.0 (0.03)	0.99 (0.03)	1.1 (0.04)	0.220
Dyslipidemia, %	21.7 (2.5)	20.6 (2.36)	25.1 (2.5)	0.187

Continuous variables were presented as means (standard error), and categorical data as percentages (standard error). SDS, standard deviation score; BMI, body mass index; WC, waist circumference; WHtR, waist-to-height ratio; SBP, systolic blood pressure; DBP, diastolic blood pressure; HDL, high-density lipoprotein; LDL, low-density lipoprotein.

**Table 2:** Proportion of participants with obesity, abdominal obesity, and hypertension according to sex.

Variable	2018	2019	2020	*p*
Male	n = 268	n = 284	n = 228	
BMI SDS	0.08 (0.09)	0.21 (0.11)	0.35 (0.11)	0.154
BMI percentile				0.179
Normal, %	79.0 (2.6)	73.5 (3.3)	69.6 (3.3)	
Overweight, %	8.2 (1.9)	8.7 (2.0)	13.4 (2.5)	
Obesity, %	12.8 (2.1)	17.8 (2.9)	17.0 (2.8)	
WC, cm	73.3 (0.7)	75.1 (0.9)	76.3 (0.8)	0.023
WC > 90 percentile, %	12.3 (2.2)	14.6 (2.4)	17.7 (2.7)	0.277
WHtR	0.44 (0.00)	0.45 (0.00)	0.46 (0.00)	0.006
WHtR > 0.5, %	15.3 (2.3)	21.9 (3.1)	24.0 (3.1)	0.073
SBP, mmHg	110 (1)	112 (1)	112 (1)	0.044
DBP, mmHg	67 (1)	69 (1)	69 (1)	0.137
SBP > 95 percentile, %	4.9 (1.4)	4.4 (1.4)	6.3 (1.5)	0.616
DBP > 95 percentile, %	4.4 (1.3)	6.3 (1.7)	8.2 (2.3)	0.275
Hypertension, %	7.1 (1.5)	9.2 (1.9)	14.2 (2.6)	0.036
Female	n = 220	n = 245	n = 183	
BMI SDS	−0.05 (0.09)	−0.02 (0.12)	0.00 (0.11)	0.925
BMI percentile				0.831
Normal, %	82.1 (2.9)	78.3 (3.5)	81.9 (2.8)	
Overweight, %	7.5 (1.7)	8.15 (2.2)	8.2 (2.0)	
Obesity, %	10.5 (2.4)	13.5 (2.7)	9.9 (2.2)	
WC, cm	67.0 (0.6)	68.2 (0.8)	68.7 (0.8)	0.172
WC > 90 percentile, %	8.6 (2.3)	10.8 (2.3)	12.8 (2.4)	0.471
WHtR	0.42 (0.00)	0.43 (0.00)	0.44 (0.00)	0.100
WHtR > 0.5, %	7.9 (2.0)	11.0 (2.6)	12.7 (2.4)	0.345
SBP, mmHg	105 (1)	105 (1)	106 (1)	0.607
DBP, mmHg	66 (1)	66 (1)	69 (1)	0.008
SBP > 95 percentile, %	2.7 (1.0)	3.3 (1.3)	4.5 (1.6)	0.613
DBP > 95 percentile, %	6.0 (1.5)	3.8 (1.7)	8.2 (2.5)	0.291
Hypertension, %	7.1 (1.5)	7.0 (2.0)	10.3 (2.7)	0.439

Continuous variables are presented as mean (standard error), and categorical data as percentages (standard error). BMI, body mass index; SDS, standard deviation score; WC, waist circumference; WHtR, waist-to-height ratio; SBP, systolic blood pressure; DBP, diastolic blood pressure.

**Table 3:** Proportion of participants with obesity, abdominal obesity, and hypertension according to age group.

Variable	2018	2019	2020	*p*
10–12 y	n = 188	n = 202	n = 162	
BMI SDS	−0.12 (0.08)	−0.05 (0.11)	0.15 (0.11)	0.141
BMI percentile				0.227
Normal, %	82.1 (2.6)	80.1 (3.7)	74.8 (3.5)	
Overweight, %	10.6 (2.2)	6.9 (2.3)	12.4 (2.7)	
Obesity, %	7.4 (2.1)	13.0 (3.0)	12.8 (2.7)	
WC, cm	65.2 (0.7)	66.0 (0.8)	67.8 (0.8)	0.058
WC > 90 percentile, %	8.1 (2.1)	8.4 (2.1)	14.1 (2.7)	0.104
WHtR	0.43 (0.00)	0.44 (0.00)	0.45 (0.01)	0.063
WHtR > 0.5, %	12.6 (2.5)	15.2 (3.3)	20.7 (3.3)	0.156
SBP, mmHg	105 (1)	106 (1)	107 (1)	0.302
DBP, mmHg	64 (1)	63 (1)	65 (1)	0.211
SBP > 95 percentile, %	6.4 (1.8)	5.0 (1.7)	7.6 (2.2)	0.623
DBP > 95 percentile, %	6.7 (1.9)	4.1 (1.6)	8.8 (2.6)	0.269
Hypertension, %	11.3 (2.3)	8.2 (2.2)	15.0 (3.1)	0.163
13–15 y	n = 159	n = 174	n = 129	
BMI SDS	0.04 (0.10)	0.15 (0.11)	0.32 (0.17)	0.359
BMI percentile				0.006
Normal, %	82.4 (3.3)	77.3 (4.0)	69.1 (4.9)	
Overweight, %	1.4 (0.77)	7.9 (2.5)	13.5 (3.7)	
Obesity, %	16.2 (3.2)	14.7 (2.9)	17.4 (3.7)	
WC, cm	70.8 (0.8)	72.5 (0.8)	74.7 (1.3)	0.034
WC > 90 percentile, %	11.3 (2.6)	12.1 (2.6)	19.9 (4.0)	0.089
WHtR	0.43 (0.00)	0.44 (0.00)	0.45 (0.01)	0.015
WHtR > 0.5, %	10.7 (2.5)	14.6 (2.8)	22.5 (4.1)	0.025
SBP, mmHg	107 (1)	109 (1)	110 (1)	0.117
DBP, mmHg	66 (1)	67 (1)	69 (1)	0.033
SBP > 95 percentile, %	0.87 (0.63)	4.9 (1.8)	8.2 (2.6)	0.007
DBP > 95 percentile, %	2.1 (1.0)	2.6 (1.1)	8.0 (2.67)	0.009
Hypertension, %	2.6 (1.1)	7.2 (2.1)	14.4 (3.5)	<0.001
16–18 y	n = 414	n = 153	n = 120	
BMI SDS	0.04 (0.08)	0.03 (0.11)	0.16 (0.10)	0.604
BMI percentile				0.765
Normal, %	79.1 (2.4)	76.2 (3.1)	77.6 (2.7)	
Overweight, %	9.1 (1.6)	8.5 (1.8)	10.33 (1.8)	
Obesity, %	11.9 (2.2)	15.2 (2.6)	12.1 (2.5)	
WC, cm	70.7 (0.7)	71.8 (0.9)	72.9 (0.8)	0.113
WC > 90 percentile, %	11.0 (1.9)	12.4 (2.2)	13.6 (2.5)	0.694
WHtR	0.44 (0.00)	0.44 (0.00)	0.45 (0.00)	0.026
WHtR > 0.5, %	12.8 (1.9)	16.2 (2.8)	16.9 (2.5)	0.425
SBP, mmHg	108 (1)	109 (1)	109 (1)	0.174
DBP, mmHg	67 (1)	68 (1)	69 (1)	0.054
SBP > 95 percentile, %	4.1 (1.1)	3.4 (1.1)	3.7 (1.2)	0.913
DBP > 95 percentile, %	4.7 (1.3)	5.6 (1.6)	7.7 (2.0)	0.409
Hypertension, %	6.8 (1.5)	8.2 (1.8)	10.5 (2.2)	0.344

Continuous variables are presented as means (standard error), and categorical data as percentages (standard error). BMI, body mass index; SDS, standard deviation score; WC, waist circumference; WHtR, waist-to-height ratio; SBP, systolic blood pressure; DBP, diastolic blood pressure.

**Table 4:** Proportion of participants with obesity, abdominal obesity, and hypertension according to BMI classification.

Variable	2018	2019	2020	*p*
Normal	n = 389	n = 410	n = 297	
WC, cm	66.7 (0.4)	67.2 (0.4)	68.1 (0.6)	0.145
WC > 90 percentile, %	0.48 (0.365)	0.00 (0.00)	0.68 (0.51)	0.149
WHtR	0.41 (0.00)	0.42 (0.00)	0.42 (0.00)	0.028
WHtR > 0.5, %	0.97 (0.48)	1.6 (0.63)	0.88 (0.53)	0.617
SBP, mmHg	106 (1)	108 (1)	108 (1)	0.214
DBP, mmHg	66 (1)	67 (1)	68 (1)	0.018
SBP > 95 percentile, %	2.8 (0.82)	2.8 (0.87)	3.5 (1.0)	0.840
DBP > 95 percentile, %	3.1 (0.90)	3.2 (0.95)	6.8 (1.9)	0.058
Hypertension, %	4.6 (1.0)	5.7 (1.20)	9.3 (2.0)	0.041
Overweight	n = 43	n = 43	n = 52	
WC, cm	79.1 (1.3)	78.3 (1.1)	80.8 (1.0)	0.196
WC > 90 percentile, %	18.5 (7.7)	9.3 (4.4)	20.1 (6.0)	0.400
WHtR	0.48 (0.00)	0.48 (0.00)	0.50 (0.00)	0.008
WHtR > 0.5, %	24.8 (8.0)	25.1 (7.0)	47.5 (7.3)	0.047
SBP, mmHg	113 (2)	111 (1)	112 (2)	0.637
DBP, mmHg	70 (1)	69 (2)	70 (1)	0.749
SBP > 95 percentile, %	11.1 (6.9)	1.2 (1.3)	4.2 (2.6)	0.097
DBP > 95 percentile, %	12.2 (5.0)	3.9 (3.7)	10.2 (5.0)	0.475
Hypertension, %	19.8 (7.4)	5.2 (3.8)	13.4 (5.4)	0.216
Obesity	n = 56	n = 76	n = 62	
WC, cm	88.9 (1.5)	90.7 (1.3)	92.7 (1.1)	0.097
WC > 90 percentile, %	74.6 (7.6)	76.3 (5.4)	92.7 (3.1)	0.031
WHtR	0.54 (0.01)	0.55 (0.01)	0.56 (0.01)	0.069
WHtR > 0.5, %	77.3 (5.5)	85.0 (5.0)	94.2 (3.1)	0.044
SBP, mmHg	114 (1)	115 (1)	116 (2)	0.434
DBP, mmHg	71 (1)	71 (1)	71 (2)	0.988
SBP > 95 percentile, %	5.8 (3.2)	10.4 (3.8)	16.9 (4.8)	0.169
DBP > 95 percentile, %	14.6 (4.4)	15.0 (5.0)	13.9 (4.9)	0.987
Hypertension, %	15.9 (4.6)	21.8 (5.5)	27.8 (5.8)	0.319

Continuous variables are presented as means (standard error), and categorical data as percentages (standard error). BMI, body mass index; WC, waist circumference; WHtR, waist-to-height ratio; SDS, standard deviation score; SBP, systolic blood pressure; DBP, diastolic blood pressure.

**Table 5:** Proportion of participants with obesity, abdominal obesity, and hypertension according to residential district.

Variable	2018	2019	2020	*p*
Urban	n = 436	n = 430	n = 350	
BMI SDS	0.01 (0.07)	0.04 (0.10)	0.16 (0.09)	0.407
BMI percentile				0.759
Normal, %	80.5 (2.1)	77.7 (3.1)	76.5 (2.5)	
Overweight, %	7.9 (1.4)	8.7 (1.5)	10.4 (1.7)	
Obesity, %	11.6 (1.8)	13.6 (2.5)	13.1 (2.1)	
WC, cm	70.4 (0.5)	71.5 (0.8)	72.7 (0.7)	0.034
WC > 90 percentile, %	10.7 (1.6)	10.7 (2.1)	14.3 (2.1)	0.319
WHtR	0.43 (0.00)	0.44 (0.00)	0.45 (0.00)	0.009
WHtR > 0.5, %	12.0 (1.7)	14.4 (2.6)	17.8 (2.2)	0.151
SBP, mmHg	108 (1)	109 (1)	109 (1)	0.133
DBP, mmHg	66 (1)	68 (1)	69 (1)	0.019
SBP > 95 percentile, %	3.7 (0.95)	3.6 (1.0)	5.8 (1.1)	0.242
DBP > 95 percentile, %	4.8 (0.96)	5.3 (1.3)	7.5 (1.6)	0.313
Hypertension, %	6.8 (1.1)	8.0 (1.5)	12.0 (1.8)	0.031
Rural	n = 52	n = 99	n = 61	
BMI SDS	0.08 (0.18)	0.44 (0.24)	0.49 (0.17)	0.216
BMI percentile				0.189
Normal, %	79.9 (6.00)	65.2 (6.3)	62.8 (5.9)	
Overweight, %	7.3 (4.4)	6.9 (4.9)	17.2 (6.3)	
Obesity, %	12.8 (4.9)	27.9 (5.0)	20.0 (4.5)	
WC, cm	69.4 (1.6)	73.6 (1.4)	74.9 (1.3)	0.021
WC > 90 percentile, %	8.7 (3.8)	25.0 (4.1)	27.1 (5.4)	0.013
WHtR	0.43 (0.01)	0.46 (0.01)	0.46 (0.01)	0.028
WHtR > 0.5, %	9.7 (3.7)	30.1 (5.4)	29.0 (5.0)	0.003
SBP, mmHg	109 (2)	108 (1)	113 (2)	0.088
DBP, mmHg	68 (2)	67 (1)	69 (1)	0.390
SBP > 95 percentile, %	5.0 (3.6)	5.8 (2.7)	3.0 (2.1)	0.777
DBP > 95 percentile, %	8.1 (4.5)	4.0 (2.5)	15.8 (7.2)	0.118
Hypertension, %	9.8 (4.6)	9.3 (3.6)	17.5 (6.9)	0.405

Continuous variables are presented as means (standard error), and categorical data as percentages (standard error). BMI, body mass index; SDS, standard deviation score; WC, waist circumference; WHtR, Waist-to-height ratio; SBP, systolic blood pressure; DBP, diastolic blood pressure.

## Data Availability

The data that support the findings of this study are available from the corresponding author upon reasonable request.
